# Coexistence of Anti-p200 Pemphigoid and Psoriasis: A Systematic Review

**DOI:** 10.3389/fimmu.2022.839094

**Published:** 2022-03-04

**Authors:** Ying-Han Xie, Si-Hang Wang, Si-Zhe Li, Ya-Gang Zuo

**Affiliations:** Department of Dermatology, State Key Laboratory of Complex Severe and Rare Diseases, Peking Union Medical College Hospital, Chinese Academy of Medical Sciences and Peking Union Medical College, National Clinical Research Center for Dermatologic and Immunologic Diseases, Beijing, China

**Keywords:** anti-p200 pemphigoid, anti-laminin γ1 pemphigoid, psoriasis, systematic review, epitope spreading

## Abstract

**Background:**

A close association between psoriasis and anti-p200 pemphigoid has been demonstrated by numerous studies. However, the clinical characteristics of patients suffering from these two entities have not yet been well-elucidated.

**Objective:**

This study aimed to review the case reports and case series, summarizing clinical features and therapeutic strategies in patients suffering from anti-p200 pemphigoid and psoriasis.

**Methods:**

A systematic review was conducted by searching PubMed, EMBASE, and Web of Science databases for studies published in English involving patients with psoriasis and anti-p200 pemphigoid on 6 September 2021. All case reports and case series reporting patients diagnosed with anti-p200 pemphigoid and psoriasis were included in this systematic review.

**Results:**

A total of 21 eligible studies comprising 26 anti-p200 pemphigoid patients with preceding psoriasis were included in the qualitative synthesis. The average age at blisters eruption was 62.5 years, and the mean duration between the two entities was 15.6 years. Twenty-four percent of patients developed bullous lesions during UV therapy. Clinical manifestation of bullae and/or vesicles was recorded in all patients, and the trunk (94.7%) was most frequently involved, with only 15.8% reporting mucosal involvement. Epitope spreading was detected by immunoblotting in 33.3% of patients. All the patients reached completed remission during the course of disease, with 36.8% experiencing at least one relapse. Monotherapy of prednisolone was the leading therapeutic approach (n=6, 31.6%) required for disease control, but 5 (83.3%) of them suffered from blister recurrence after tapering or ceasing corticosteroid.

**Conclusion:**

Most of the clinical aspects of patients with anti-p200 pemphigoid and psoriasis were similar to what was demonstrated in previous articles on anti-p200 pemphigoid. Nevertheless, compared with other anti-p200 pemphigoid cases without psoriasis, a clinical manifestation pattern with more frequent involvement of the trunk and less mucosal involvement was illustrated in those with psoriasis. Generally, monotherapy is sufficient for a complete remission for such patients. However, one or more relapses have been recorded in a considerable portion of patients, especially those prescribed with prednisolone. It reminded us to be more cautious during a tapering of medication.

## Introduction

Anti-p200 pemphigoid, a rare subset of autoimmune bullous disease (AIBD), was initially reported in 1996 by Japanese researchers (1, 2). Autoantibodies against a 200-kDa dermal protein, which is localized in the lower lamina lucida of the basement membrane zone (BMZ), is the defining characteristic of this disease. With 90% of sera from anti-p200 pemphigoid patients showing immunoreactivity to γ-1 chain of laminin, some investigators suggested to rename this disease as anti-laminin γ1 (lamγ1) pemphigoid (3). Due to its rarity and unavailable detective technology in most countries, the prevalence of anti-lamγ1/p200 pemphigoid has not been classified. According to the study conducted by Dainichi et al., a sizable portion of patients with anti-lamγ1 pemphigoid could have been misdiagnosed as epidermolysis bullosa acquisita (EBA) (4). Additionally, in recently published studies, anti-lamγ1 pemphigoid was considered to be the most common floor-binding subepidermal AIBD, of which detection by indirect immunofluorescence (IIF) microscopy on human salt-split skin (SSS) illustrated serum autoantibodies targeting at the dermal side of split (5–7). Moreover, a clear male predominance of approximately 75% of 113 patients with anti-lamγ1 pemphigoid was summarized by Kridin et al. (8, 9). The mean age of anti-lamγ1 pemphigoid patients was 65.5 years (range 5–94) (9), younger than those with bullous pemphigoid (BP).

Anti-lamγ1 pemphigoid patients always present with bullae, urticarial plaques, and occasional pruritus, which may mimic other subepidermal AIBDs, especially BP (10). The mucosal lesion was reported in 20% to 40% of patients (9, 11). Histologically, perilesional biopsy specimen shows a dermal-papillary infiltrate pattern, commonly containing neutrophils, and/or occasionally eosinophils, along the dermo-epidermal junction (3, 8, 11). However, it is insufficient for the differentiation of anti-lamγ1 pemphigoid from other subepidermal AIBDs with the result of histopathology alone (12). Regarding the immunopathological and serologic characterization of anti-lamγ1 pemphigoid, detection of autoantibodies against the 200-kDa dermal protein by immunoblotting (IB) is the golden standard for the diagnosis of anti-lamγ1 pemphigoid. However, the extraction procedure of dermal protein was only available in specific research centers (3). Recently, a novel two-step IB assay was proposed by Solimani et al., which utilized recombinant lam111, recombinant lam411 and purified lamγ1 in place of dermal extractions for the detection of anti-lamγ1 autoantibodies, ulteriorly improved the overall sensitivity to 98.2% (13). This study may provide a new IB mode for detecting anti-lamγ1 pemphigoid, and facilitate the diagnosis of this disease worldwide to a certain degree. Apart from this, several researchers attempted to apply the serration pattern analysis to the classification of AIBDs (7, 14, 15), which remains to be verified in larger populations.

So far, no standard therapeutic approaches for anti-lamγ1 pemphigoid have been defined. Compared to EBA and BP, a more benign course was observed in patients with anti-lamγ1 pemphigoid (11). Britva et al. summarized the treatment strategies administrated to 113 patients. They proposed that approximately 40% of patients experienced a relapsing course, and a combination therapy of oral corticosteroids and immunomodulators was the most commonly adopted treatment strategies for anti-lamγ1 pemphigoid (16).

It is declared that anti-lamγ1 pemphigoid patients often have preexisting psoriasis. According to a recent systemic review, the prevalence of psoriasis in Japanese cases and non-Japanese cases with anti-lamγ1 pemphigoid was 56.0% and 6.4%, respectively (9). Given the close association between anti-lamγ1 pemphigoid and psoriasis, it is rational to suspect the development of anti-lamγ1 pemphigoid in psoriasis patients when bullae are observed. However, the mechanism underlying the frequent coexistence of these two entities and the clinical characteristics of these patients have not yet been well-elucidated. Other complications in anti-lamγ1 pemphigoid patients included malignancy, ulcerative colitis, renal disease, neurological disorders, and several cutaneous diseases (e.g., autosomal recessive congenital ichthyosis, scabies, and acquired perforating dermatosis) (9, 17–22). Interestingly, a parallel disease course was reported in 2 ulcerative colitis patients (17, 20). In this study, we reviewed the available epidemiological, clinical, histological, immunopathological, and therapeutic data of patients diagnosed with psoriasis and anti-lamγ1 pemphigoid, attempting to summarize multiple clinical features of such patients that could help make an earlier diagnosis and perform suitable treatments.

## Method

A primary literature review was performed on three separate online databases (PubMed, EMBASE, and Web of Science). We identified eligible articles from database inception to 6 September 2021. We also screened the references of included studies, in order to expand the search scope of literature. An additional literature review on lamγ1 pemphigoid was conducted for a further statistical evaluation on several criteria between the cases with psoriasis and the cases without. The detailed search strategy is included in **Supplementary Files 1** and **2**.

### Selection Criteria

Studies reporting one or more patients with a diagnosis of anti-lamγ1 pemphigoid and psoriasis were all included. Criteria for the diagnosis of anti-lamγ1 pemphigoid were listed below:

(i) clinical manifestation: similar to subepidermal AIBD patients;

(ii) histopathology: a picture of subepidermal blister;

(iii) DIF: a linear IgG and/or C3 deposition pattern along the dermo-epidermal junction;

(iv) IB analysis: a 200-kDa band detected with patients’ sera on human dermal extracts;

(v) exclusion of other subepidermal AIBDs.

It was excluded if the manuscripts were published in the following types: abstracts, conference presentations, editorials, reviews, and expert opinions. Articles in language other than English were ruled out as well. All abstracts were reviewed by reviewers. Only those with full-text documents were ultimately selected for eligibility.

### Data Extraction

Two investigators completed data extraction independently. When there was a divergence of opinion, we would discuss this disagreement and settle it by consensus. The following variables were gathered as available: age, sex, location, morphological features, histopathology, immunopathology, comorbidities, therapeutic strategies, length of follow-up, and clinical outcomes.

### Data Analysis

It was unfortunate that relevant data regarding characteristics to be analyzed were only available for certain cases. Thus, percentages referred to the total number of patients for whom a specific outcome was available. Analyses were performed on SPSS version 26 (SPSS, Chicago, IL, USA).

## Result

**Figure 1:**
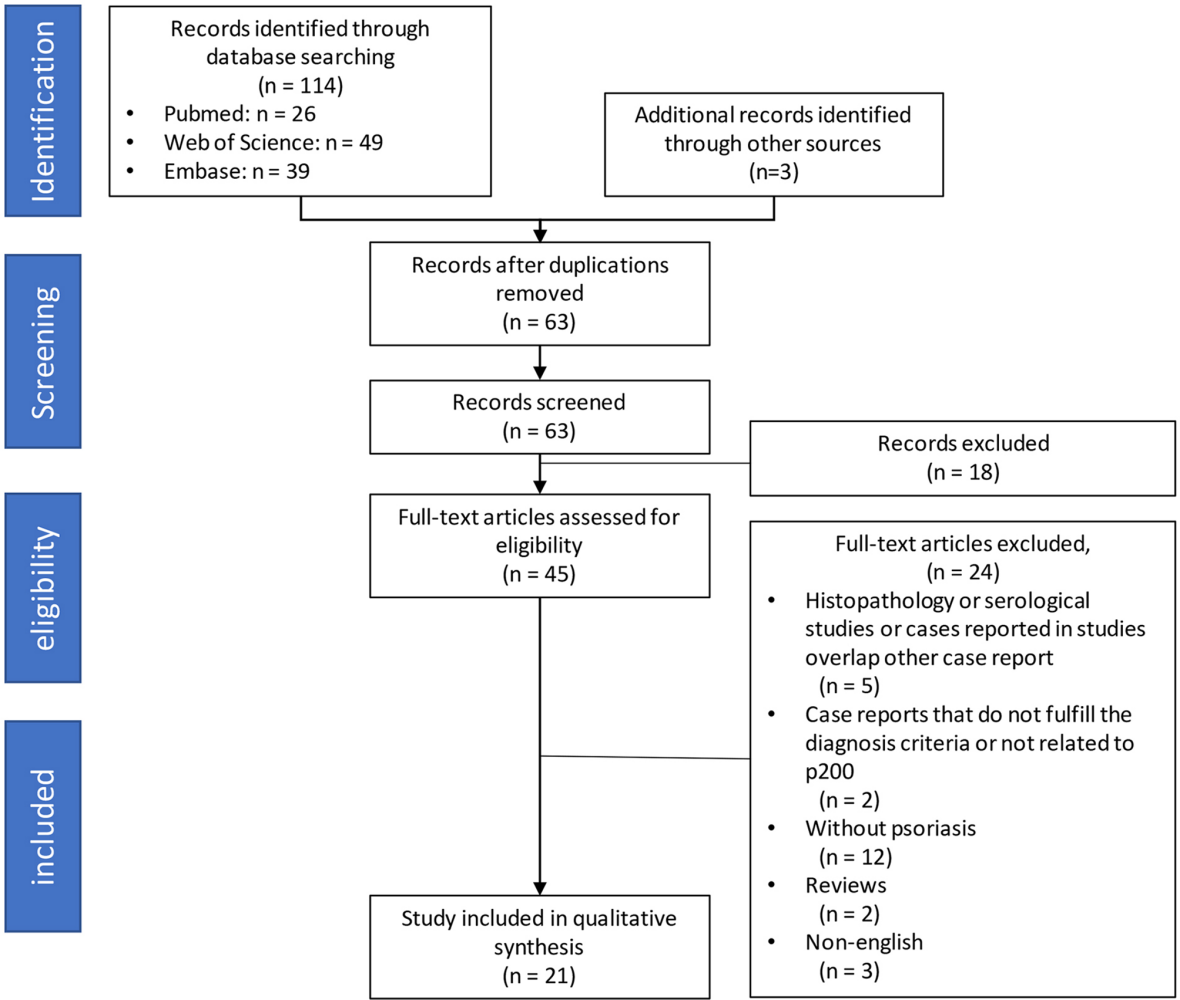
Flow chart for the process of article screening and selection on three databases.

A total of 114 studies were identified through the online literature database search. Other sources yielded three additional articles. After the removal of duplicates, 63 remaining manuscripts underwent the screening process for titles and abstracts. During this procedure, irrelevant studies and cases which did not satisfy our inclusion criteria were excluded. Full-text examination was conducted in 45 remaining studies. Eventually, 21 case reports, published between 1996 and 2021, were included in the qualitative synthesis. The Preferred Reporting Items for Systematic Reviews and Meta-Analyses (PRISMA) flow diagram is presented in **Figure 1**.

**Table 1** summarized the characteristics of the included cases from the systematic search. Concomitant psoriasis was reported in 26 patients, and 23 of them were described by Japanese researchers. And 93 patients were reported to be diagnosed with anti-lamγ1 pemphigoid without psoriasis, indicating that 21.8% of anti-lamγ1 pemphigoid cases were complicated with psoriasis. Psoriasis vulgaris (PsV, n = 21) was the most prevalent type of psoriasis, followed by pustular psoriasis (PP, n = 4) (23–26) and erythrodermic psoriasis (n = 1, **Table 1**) (27). Two patients with PP (26, 28) and one with erythrodermic psoriasis (27) had a previous PsV history. Among patients with PP and anti-lamγ1 pemphigoid, blisters appeared in two patients after UV therapy with an interval of three weeks (28) and three years (23) from PP onset, respectively, distinguishing from a general long duration between psoriasis and anti-lamγ1 pemphigoid.

**Table 1:** Demographic characteristics of the reported anti-lamγ1 pemphigoid patients complicated with psoriasis.

		*N (%)* or mean (± SD)	Number of cases for which data were available
**N total**		26	
	Males	23 (88.5%)	26
	Females	3 (11.5%)	26
	Age of anti-p200 pemphigoid (years)	62.5 (12.7)	26
	Age of psoriasis onset (years)	47.6 (17.1)	19
**Duration from psoriasis to p200 (years)**		15.6 (11.1)	19
	0-9	8 (42.1%)	
	10-19	2 (10.5%)	
	20-29	4 (21.1%)	
	>29	5 (26.3%)	
**Associated triggers**			21
	Psoralen UVA therapy	3 (14.3%)	
	Narrow band UVB therapy	2 (9.5%)	
	Discontinuation of drugs	3 (14.3%)	
	Stress events	1 (4.8%)	
	Worsen ulcerative colitis	1 (4.8%)	
	dipeptidyl peptidase-4 inhibitor	1 (4.8%)	
**Types of psoriasis**			26
	Psoriasis vulgaris	21 (80.8%)	
	Pustular psoriasis	4 (15.4%)	
	Erythrodermic psoriasis	1 (3.8%)	

N, number; SD, standard deviation.

The male patients (n = 23, 88.5%) outnumbered female ones (n = 3, 11.5%) by 7.7:1 in anti-lamγ1 pemphigoid concomitant psoriasis patients, which suggested a more evident male predilection than patients without psoriasis(2.6:1), and previous research on patients with psoriasis and AIBD (5.7:1) (29). The overall patient age (mean ± SD) at anti-lamγ1 pemphigoid onset was 62.5 ± 12.7 years (range, 31-82 years), and psoriasis onset 47.6 ± 17.1 years (range, 31-82 years). The mean age (mean ± SD) of blister eruption in them was comparable with that in patients without psoriasis (66.7 ± 16.3, *P* = 0.221) and significantly younger than patients with BP (8). The overall duration (mean ± SD) from psoriasis to the onset of pemphigoid was 15.6 ± 11.1 years (range, 26-81 years), in which eight (42.1%) patients developed pemphigoid during the first decade after the diagnosis of psoriasis. Among the five (23.8%) patients who developed bullous lesions during UV therapy, three were performed with psoralen UVA therapy (23, 30, 31), and two were on the treatment session of narrowband UVB (28, 32). In addition, sudden cession of drugs (25, 26, 33), stress event (34), dipeptidyl peptidase-4 inhibitor (35), and ulcerative colitis (20) could also be possible triggers leading to pemphigoid in psoriasis patients.

**Figure 2:**
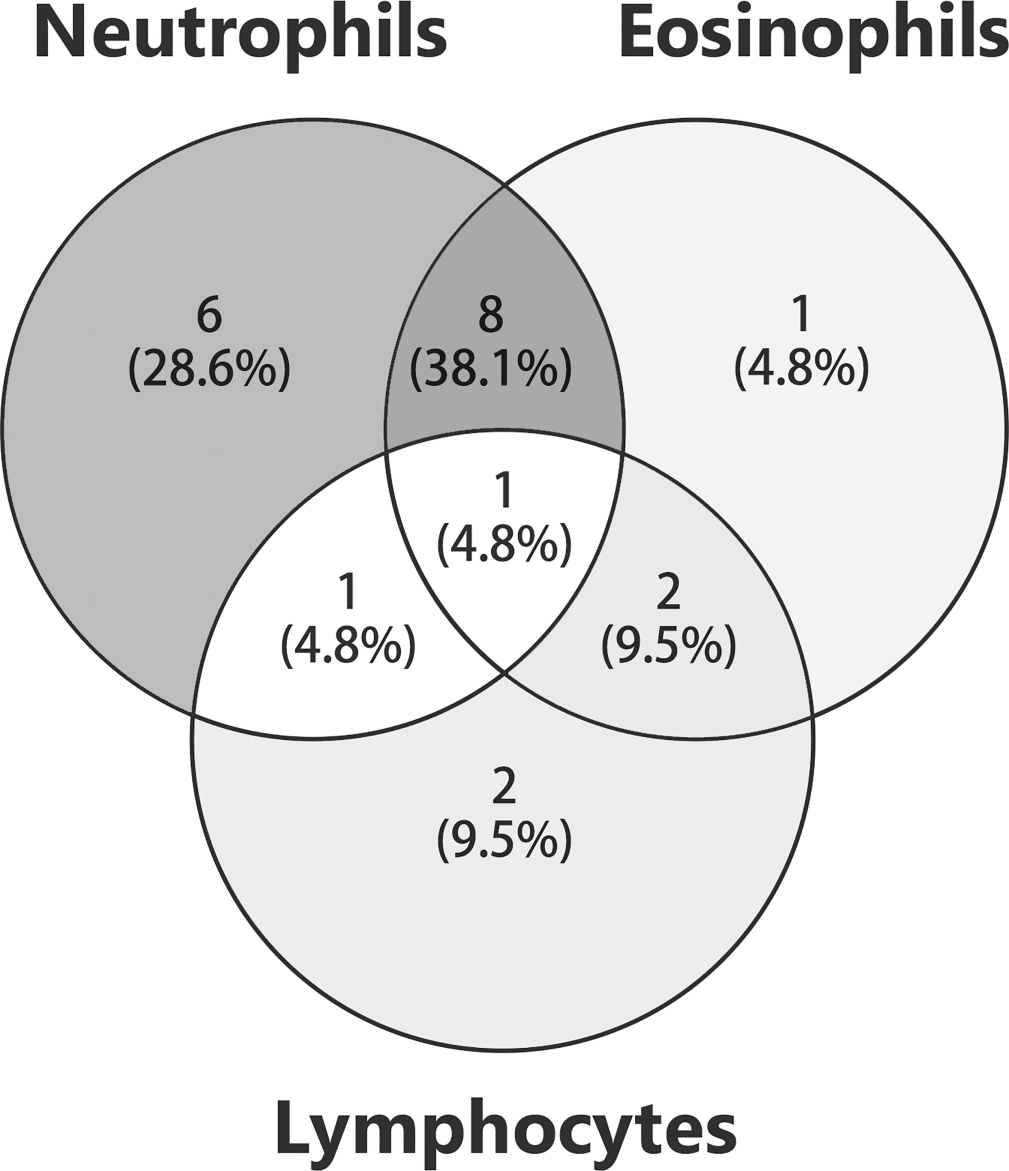
Histology in patients with anti-p200 pemphigoid and psoriasis. The numbers of patients with different inflammatory cell infiltration were shown. The overlaps of circles indicated that patients were detected with infiltration patterns of mixed inflammatory cells.

Seventeen cases reported the distribution of psoriatic lesions when patients were admitted to the hospital. An entire-body involvement was recorded in seven cases, followed by the distribution of both trunk and extremities (n = 6). Interestingly, one patient had no classical psoriatic appearance, apart from onycholysis of the toe nails (36). For pemphigoid lesions, clinical manifestation of bullae and/or vesicles was reported in all cases **(** **Table 2** **)**. 28.6% (6/21) of patients had edematous erythema. 9.5% (2/21) had pustules. 14.3% (3/21) developed scars and/or milia after treatment. The lesion distribution was announced in 19 cases. The trunk was the most commonly involved area (94.7% vs. 67.2% in non-psoriasis patients, *P* = 0.017), followed by extremities (78.9%), palmoplantar (36.8%), and cephalic (31.6%). Involvement of both trunk and extremities was reported in seven (36.8%) patients. Five (26.3%) patients suffered from cutaneous erosions on the whole body. Two cases had genital involvement, and only one case (10.6%) had oral mucosa involvement, which were lower than those without psoriasis (34.9%; *P* = 0.010). A slightly lower frequency of mucosal involvement was recorded in concomitant psoriasis compared to non-psoriasis patients (15.8% vs. 38.6%). An infiltrate of both neutrophils and eosinophils in upper dermis was recorded in eight (38.1%) patients, and infiltrate of neutrophils in six (28.6%) patients **(** **Figure 2** **)**. Interestingly, two cases (9.5%) reported an infiltrate pattern of lymphocytes, which was absent in the patients without psoriasis (*P =* 0.036, **Table 3**).

**Table 2:** Clinical manifestation and distribution of lesions in reported cases.

		Anti-p200 pemphigoid cases with psoriasis *N (%)* 95% *CI*	Anti-p200 pemphigoid cases without psoriasis *N (%)* 95% *CI*	*P Value*
**Morphology of cutaneous lesions**				
	Bullae/vesicles	21 (100%) -	92 (100%) -	–
	Erythematous plaques	6 (28.6%) 0.075-0.496	34 (37.0%) 0.269-0.470	0.468
	Pustules	2 (9.5%) -0.042-0.232	0 (0%) -	0.033
	Scars/milia	3 (14.3%) -0.020-0.306	12 (13.0%) 0.060-0.201	1.000
**Distribution of lesions^#^**				
	Extremities	15 (78.9%) 0.588-0.991	56 (83.6%) 0.745-0.927	0.899
	Trunk	18 (94.7%) 0.837-1.058	45 (67.2%) 0.566-0.787	0.017
	Palms and soles	7 (36.8%) 0.130-0.607	40 (59.7%) 0.476-0.718	0.077
	Head and neck	6 (31.6%) 0.086-0.546	22 (32.8%) 0.213-0.444	0.714
	Entire body	5 (26.3%) 0.045-0.481	12 (17.9%) 0.085-0.273	0.515
	Mucosal involvements^$^	3 (15.8%) -0.023-0.338	32 (38.6%) 0.279-0.492	0.059
**Mucosal involvements^$^**				
	Nasal mucosa	0 (0%) -	4 (4.8%) 0.001-0.095	1.000
	Conjunctiva	0 (0%) -	5 (6.0%) 0.008-0.113	0.581
	Oral mucosa	1 (5.3%) 0.058-0.163	29 (34.9%) 0.245-0.454	0.010
	Genital mucosa	2 (10.6%) 0.047-0.257	11 (13.3%) 0.058-0.207	1.000

N, number; CI, Confidence interval.

^#^Data of 19 and 67 cases were available in patients with and without psoriasis, respectively.

^$^Data of 19 and 83 cases were available in patients with or without psoriasis, respectively.

**Table 3:** Histology feature in reported cases.

		Anti-p200 pemphigoid cases with psoriasis *N (%) 95% CI*	Anti-p200 pemphigoid cases without psoriasis *N (%) 95% CI*	*P Value*
Histology*				
	Neutrophils	6 (28.6%) 0.075-0.496	28 (32.2%) 0.222-0.422	0.749
	Eosinophils	1 (4.8%) -0.052-0.147	11 (12.6%) 0.075-0.496	0.519
	Lymphocytes	2 (9.5%) 0.042-0.232	0 (0%) -	0.036
	Neutrophils/eosinophils	8 (38.1%) 0.033-0.167	36 (41.4%) 0.308-0.519	0.783
	Lymphocytes/eosinophils	2 (9.5%) 0.042-0.232	4 (4.6%) 0.001-0.091	0.723
	Lymphocytes/neutrophils	1 (4.8%) -0.052-0.147	1 (1.1%) -0.011-0.034	0.353
	Lymphocytes/eosinophils/neutrophils	1 (4.8%) -0.052-0.147	4 (4.6%) 0.001-0.091	1.000
	Not detected	0 (0%) -	3 (3.4%) 0.005-0.074	1.000

N, number; CI, Confidence interval.

*Data of 21 and 87 cases were available in patients with and without psoriasis, respectively.

Results of DIF and SSS-IIF were demonstrated in 24 cases. DIF showed a linear deposition pattern of IgG and/or C3 along the BMZ in all patients except one with the deposition of IgG alone. On SSS-IIF, an exclusively floor-binding picture was recorded in 18 cases, both roof- and floor-binding pattern in six patients (20, 25, 30, 32, 33, 36). IB analysis showed autoantibodies against the BP180 C-terminal domain alone, BP230 alone slightly, BP260 alone, BP180 and BP230 collectively, and both BP180 and laminin-332 were detected in 2, 2, 1, 1, and 1 patient respectively (20, 25, 30, 32, 33, 36) **(** **Table 4** **)**.

**Table 4:** Immunopathological and serologic characterization of reported cases.

		Anti-p200 pemphigoid cases with psoriasis *N (%) 95% CI*	Anti-p200 pemphigoid cases without psoriasis *N (%) 95% CI*	*P Value*
**IIF of SSS^#^**				
	D (+)	18 (75.0%) 0.563-0.937	69 (84.2%) 0.761-0.922	0.468
	E (±)*, D (+)	2 (8.3%) 0.036-0.203	2 (2.4%) -0.010-0.058	0.220
	E (+), D (+)	4 (16.7%) 0.006-0.327	11 (13.4%) 0.059-0.209	0.945
**IB analysis**				
	200-kDa protein	21 (100%) -	82 (100%) -	–
	BP180	2 (9.5%) -0.042-0.232	1 (1.2%) -0.012-0.036	0.105
	Bp230	2 (9.5%) -0.042-0.232	7 (8.5%) 0.024-0.147	1.000
	BP260 (a variant of EBA)	1 (4.8%) -0.052-0.147	1 (1.2%) -0.012-0.036	0.368
	BP290	0 (0%) -	2 (2.4%) -0.010-0.058	1.000
	Laminin-332	0 (0%) -	5 (6.1%) 0.008-0.114	0.554
	BP180 and BP230	1 (4.8%) -0.052-0.147	2 (2.4,-0.010-0.058	0.499
	BP180 and laminin-332	1 (4.8%) -0.052-0.147	1 (1.2%) -0.012-0.036	0.368
	BP230 and laminin-332	0 (0%) -	1 (1.2%) -0.012-0.036	1.000

SD, Standard Deviation; CI, Confidence interval; D, dermal; E, epidermal; BP, bullous pemphigoid; EBA, epidermolysis bullosa acquisita; IB, immunoblotting; IIF, indirect immunofluorescence; SSS, salt-split skin.

*: ± indicated those with an equivocal result of IIF.

^#^Data of 24 and 82 cases were available in patients with and without psoriasis, respectively.

Treatments for the previous psoriasis were disclosed in 16 patients, with topical corticosteroids most commonly prescribed, apart from the one who was treated with oral prednisolone for the eruption of pustules over her whole body (26). Six patients had a history of UV therapy, but one patient was not on the treatment session at the onset of pemphigoid (33) **(** **Table 5** **)**. Among 19 patients who reported therapy strategies leading to first complete remission, six patients were treated with prednisolone (20, 26, 31, 33, 37, 38), three patients cyclosporine (1, 25, 30) and the rest minocycline (30), topical corticosteroids (23), and dapsone (34), respectively, as effective monotherapy. The remaining seven (36.8%) patients were administrated with corticosteroid (orally or topically) and other adjuvant therapies [cyclosporine (28), mycophenolate mofetil (39), plasmapheresis (32, 40), apremilast (35), and intravenous immunoglobulin (IVIG) (36)]. During an average follow-up duration (mean ± SD) of 18.9 ± 11.4 months, all cases demonstrated clinical remission of anti-lamγ1 pemphigoid, with seven patients suffering from at least one relapse of blisters during tapering or ceasing of medication, six of which occurred in systemic prednisolone prescribed cases **(** **Table 6** **)** (20, 26, 31, 33, 38, 40). With respect to psoriasis, nine out of 15 patients (60%) reported a complete remission, while mild psoriatic plaques flared up in five patients (33.3%) during follow-up, which generally responded well with topical corticosteroid.

**Table 5:** Therapy strategies for psoriasis before blister formation.

Treatment	*N* (%)
Prednisolone	1 (6.25%)
Topical corticosteroid	5 (31.25%)
Topical corticosteroid, vitamin D3	4 (25%)
Topical corticosteroid, psoralen UVA	2 (12.5%)
Topical corticosteroid, tar, psoralen UVA	1 (6.25%)
Topical corticosteroid, narrow band UVB	1 (6.25%)
Topical corticosteroid, topical vitamin D3, narrow band UVB, etretinate	1 (6.25%)
Topical corticosteroid, topical vitamin D3, narrow band UVB, etretinate, ciclosporin	1 (6.25%)
**Total**	16 (100%)

N, number.

**Table 6:** Treatment tactics of reported patients with psoriasis and anti-lamγ1 pemphigoid.

Therapy	*N (%)*	Complete remission	Remission with at least one relapse
**Monotherapy**	12 (63.2)		
Minocycline		1	0
Topical corticosteroids		1	0
Cyclosporine		3	0
Systemic PSL		1	5
Dapsone		0	1
**Corticosteroids + adjuvant therapy**	7 (36.8)		
Systemic PSL and MMF		1	0
Systemic PSL and cyclosporine		2	0
Systemic PSL, IVIG and other adjuvant therapy		1	0
Systemic PSL, plasmapheresis and other adjuvant therapy		1	1
Minocycline, topical corticosteroid, apremilast		1	0
**Total**	19	12 (63.2)	7 (36.8)

N, number; PSL, prednisolone; MMF, Mycophenolate Mofetil; IVIG, Intravenous immunoglobulin.

## Discussion

This systematic review summarized clinical features and therapeutic strategies utilized in anti-lamγ1 pemphigoid patients with a preceding psoriasis history, most reported from Japan. Even with a mean duration of 15.6 ± 11.1 years (range, 26-81 years), a sizeable portion (42.1%) of patients developed AIBD in the first decade after psoriasis onset. Bullae and vesicles were the predominant clinical manifestation of these patients, coupled with a lower prevalence of mucosal involvement compared with other anti-lamγ1 pemphigoid patients. Autoantibodies against the BMZ antigens other than 200-kDa dermal protein were detected by IB in 33.3% of patients. All the patients have reached a completed remission, with a considerable portion of patients (36.8%) having experienced at least one relapse. Most of the relapses occurred during the tapering course of prednisolone.

This review indicated that 21.8% of anti-lamγ1 pemphigoid cases were complicated with psoriasis, slightly lower than the incidence of 28-48% estimated in previously published literature (9, 41). In this study, the mean interval of the two entities was 15.6 years, which was in line with what was reported by Krindin et al. (29), who illustrated that the average duration from the onset of psoriasis to the development of AIBD was 14.6 years. With the overall prevalence of psoriasis in adults ranging from 0.1% in east Asia to 1.5% in western Europe (42), it could be inferred that an intimate association exists between psoriasis and anti-lamγ1 pemphigoid. PsV was the most common type of psoriasis to be complicated, and quite a fair share of the cases of erythrodermic psoriasis and PP had a prior PsV history. Moreover, four (15.4%) patients had concomitant PP. A previous survey on Japanese psoriasis patients demonstrated that only 137 out of 104,669 patients (1.3%) were diagnosed with PP (43), suggesting a much closer association between PP and anti-lamγ1 pemphigoid.

A quite similar morphology of cutaneous lesions was recorded in anti-lamγ1 pemphigoid patients with or without psoriasis. Bullae/vesicles were the universal clinical manifestation in those patients. Occasionally, erythematous plaques were also recorded. It seemed that pustules could be a specific manifestation in anti-lamγ1 pemphigoid patients complicated with psoriasis. However, it should be derived from concomitant PP rather than anti- lamγ1 pemphigoid. Distinct from other anti- lamγ1 pemphigoid cases, patients with psoriasis always presented with a lesional distribution on the trunk rather than extremities. Furthermore, mucosal lesions were not as universal as those reported in non-psoriasis patients, and those discovered in previous studies on anti-lamγ1 pemphigoid (9, 44, 45). Additionally, unlike a predominantly oral erosion feature in other anti-lamγ1 pemphigoid cases (9, 44), the genital area could be more frequently involved in anti-lamγ1 pemphigoid patients complicated with psoriasis.

Recently, Holtsche et al. conducted a study on serologic characterization of anti-lamγ1 pemphigoid, which indicated that epitope spreading (ES) was detected in 39.2% serum of the patients, including laminin 332, BP180, BP230, and type VII collagen (46). Previous research on psoriasis has also revealed the presence of antibodies targeting laminin 1 and type IV collagen in both uninvolved and involved psoriatic skin (47). Patients with anti-lamγ1 pemphigoid and psoriasis had identical ES ratio (33.3%, by IB analysis) as recorded in previous studies. Despite detection of antibodies against the BMZ protein other than 200 kDa dermal protein or lamγ1, no mucosal involvement (25), rapid response to systemic treatments, and non-scarring resolution of skin lesions (20, 32, 37) are helpful for the exclusion of other AIBDs. Except for one patient with palmoplantar and lower limbs erosions, patients detected with ES phenomenon had an evident entire-body involvement pattern (n=6). However, even with a widespread lesion manifestation, those patients still presented with a benign prognosis. According to studies on ES in BP patients, a parallel association between ES and severity of BP was demonstrated. In contrast, no such explorations have been conducted in anti-lamγ1 pemphigoid patients. In 2012, Monshi et al. (48) conducted a long-term study on the IB result of a patient with anti-lamγ1 pemphigoid and proposed that ES was related to insufficient therapy to pemphigoid or long disease duration.

No standard therapeutic strategies have been defined for anti-lamγ1 pemphigoid (11). Britva et al. indicated that anti-lamγ1 pemphigoid might not be so benign a disease as previously estimated, for 39.6% of patients had a relapsing course, and a sizable portion of patients should be treated with systemic corticosteroid and adjuvant immunomodulatory agents for controlling disease (16). Distinct from anti-lamγ1 pemphigoid cases, a large portion of patients with concomitant psoriasis could reach disease control with monotherapy. Systemic prednisolone was the most common agent, and an average dose of 40~60 mg/d was required for disease control, not as low as expected. A similar recurrence rate (36.8%) was recorded in anti-lamγ1 pemphigoid and psoriasis patients. Moreover, most of the relapses occurred in patients administrated with prednisolone as monotherapy during tapering of medication, for whom a final disease control was reached with an addition of multiple adjuvant therapies. Additionally, limited article implied that a significant portion of patients still had flares of psoriatic lesions after remission of pemphigoid, who were generally treated with topical corticosteroids.

Despite the fact that an intimate association between psoriasis and anti-lamγ1 pemphigoid seems to be inferred, no *in vivo* or *in vitro* studies have been conducted to elucidate it. Several hypotheses have been proposed. It has been hypothesized that metalloproteinase-9 (MMP-9) played an essential role in the pathogeneses of anti-lamγ1 pemphigoid (4). In psoriasis patients, overproduction of multiple cytokines, for instance, TNF-α, IL-1, and IFN-γ, was expected to result in an increased expression of MMP-9. Furthermore, MMP-9 released from neutrophils in psoriasis patients could stimulate degradation of laminins (3, 49). As a result, exposure of laminin fragments would induce the production of various autoantibodies targeting the BMZ proteins. Besides, the destruction of BMZ can further promote the development of psoriasis due to increased keratinocyte instability and proliferative tendency (29). Another presumed mechanism is that UV therapy conducted for psoriasis could result in a configuration change of the BMZ protein (29, 50). Washio et al. postulated that the BMZ antigenicity alteration and antigen exposure caused by UV irradiation could decrease the threshold of spontaneous generation of antibodies targeting the BMZ proteins (50). With six of our patients having a history of UV therapy and five of them exactly in the treatment session when there was an eruption of blisters, we should pay special attention to the pathogenesis of UV irritation in anti- lamγ1 pemphigoid patients complicated with psoriasis.

The main limitation of our review is that the data were extracted from case reports or case series. Selection bias could inevitably exist in these articles, and information provided is occasionally brief, scarce, and not comprehensive. For instance, most cases focused on the clinical aspects of anti-lamγ1 pemphigoid. However, records on psoriasis were limited or missing, resulting in enormous difficulties in describing the clinical features of psoriasis. Thus, this study did not describe in detail about the clinical characteristics of psoriasis associated with anti-p200 pemphigoid. Additionally, most cases were reported from Japan (88.5%). However, with only three non-Japanese cases identified during our literature review, it was insufficient to perform a comparison between Japanese and non-Japanese anti-lamγ1 pemphigoid patients to explore the potential factors. Concerning the existence of racial or regional difference in those patients, we speculated that it was because that Japan had the longest research history on anti-lamγ1 pemphigoid and was equipped with a commercially available kit for the detection of autoantibodies targeting at lamγ1. To confirm this assumption, the characteristics of this disease remain to be verified on a worldwide basis. Moreover, most articles did not apply severity scores of psoriasis and AIBD. Consequently, quantified and systematic descriptions of the disease course were unavailable in these patients. Lastly, with no expert consensus on therapeutic strategies for such patients, multiple agents and variable treatment doses were administrated to different ones, making it quite challenging to conduct a transverse comparison among different medication strategies.

In conclusion, this systematic review summarized multiple clinical and therapeutic aspects of patients with anti-lamγ1 pemphigoid and psoriasis. With an average interval of more than ten years, tense blisters could be observed in those patients with preceding psoriasis. Interestingly, the duration of patients with PP could be shorter than those with other types of psoriasis. Compared to other anti-lamγ1 pemphigoids without psoriasis, a more frequent distribution on the trunk and less mucosal involvement were recorded in our review. The majority of these patients could reach clinical remission with monotherapy. However, special attention should be paid during the tapering course of medications in case of blisters recurrence, especially those administrated with prednisolone. Further researches are required to substantiate these findings in other regions and racial groups.

## Data Availability Statement

The original contributions presented in the study are included in the article/**Supplementary Material**. Further inquiries can be directed to the corresponding author.

## Author Contributions

Y-HX extracted and analyzed the data, wrote the manuscript, and prepared the figures and tables. S-HW extracted the data and revised the manuscript. S-ZL revised the manuscript and extracted the data of anti-p200 pemphigoid patients without psoriasis. Y-GZ acquired the fundings, instructed and revised the manuscript. All authors contributed to the article and approved the submitted version.

## Funding

This study was supported by the National Natural Science Foundation of China (grant number 81972944) and the Beijing Natural Science Foundation (grant number 7192166).

## Conflict of Interest

The authors declare that the research was conducted in the absence of any commercial or financial relationships that could be construed as a potential conflict of interest.

## Publisher’s Note

All claims expressed in this article are solely those of the authors and do not necessarily represent those of their affiliated organizations, or those of the publisher, the editors and the reviewers. Any product that may be evaluated in this article, or claim that may be made by its manufacturer, is not guaranteed or endorsed by the publisher.

## Abbreviations

lamγ1, laminin γ1; IB, immunoblotting; PsV, Psoriasis vulgaris; PP, pustular psoriasis; ES, epitope spreading.
