# Supplementary material for: Coexistence of Anti-p200 Pemphigoid and Psoriasis: A Systematic Review
Source: Front Immunol. 2022 Mar 4;13:839094. doi: 10.3389/fimmu.2022.839094 (PMC8934418; doi:10.3389/fimmu.2022.839094)
Supplement: Supplementary file 2 [file DataSheet_2.docx]

**Supplementary File 2** Detailed search strategy on three databases for anti-p200 pemphigoid cases

| **PubMed** | |
| --- | --- |
| #1 | **"Anti-p 200" OR Anti-p200** |
| #2 | **(p200) OR ("p 200") OR (p-200)** |
| #3 | laminin gamma 1[Supplementary Concept] |
| #4 | "laminin gamma 1" OR "Laminin gamma1" |
| #5 | "Anti-laminin gamma 1" **OR** "Anti-laminin gamma1" **OR** "Antilaminin gamma1" **OR** "Anti-laminin gamma-1" **OR** "Anti-laminin-gamma1" **OR** "Antilaminin-gamma1" **OR** "Anti-laminin-gamma 1" **OR** "Anti-laminin-gamma-1" |
| #6 | (LAMC1-protein) OR (LAMC1 protein) OR LAMC1 |
| #7 | pemphigoid |
| #8 | "dermal" and ("200 kD" or "200 kda") and "antigen" |
| #9 | (#1) OR (#2) OR (#3) OR (#4) OR (#5) OR (#6) |
| #10 | (#9) AND (#7) |
| #11 | (#10) OR (#8) |
| **EMBASE** | |
| #1 | **'pemphigoid'/exp OR 'pemphigoid'** |
| #2 | **"Anti p 200" OR "Anti p200"** |
| #3 | **p200 OR "p 200"** |
| #4 | **"**laminin gamma 1**"** OR **"**Laminin gamma1**"** or **"**laminin γ 1**"** OR **"**Laminin γ1**"** |
| #5 | 'antilaminin gamma 1' OR 'antilaminin gamma1' OR 'antilaminin γ 1' OR 'antilaminin γ1' OR 'anti laminin gamma 1' OR 'anti laminin gamma1' OR 'anti laminin γ 1' OR 'anti laminin γ1' |
| #6 | 'lamc1' |
| #7 | 200 AND (kd OR kda) AND dermal AND antigen |
| #8 | #2 OR #3 OR #4 OR #5 OR #6 |
| #9 | #1 AND #8 |
| #10 | #7 OR #9 |
| **Web of Science** | |
| #1 | TI=((p200 OR "p 200" OR p-200) AND pemphigoid) OR TS=((p200 OR "p 200" OR p-200) AND pemphigoid) OR AB=((p200 OR "p 200" OR p-200) AND pemphigoid) |
| #2 | TI=((Anti-p200 OR "Anti p200" OR "Anti p-200" OR Anti-p-200 OR "Anti p 200"OR "Anti-p 200" ) AND pemphigoid) OR TS=((Anti-p200 OR "Anti p200" OR "Anti p-200" OR Anti-p-200 OR "Anti p 200"OR "Anti-p 200" ) AND pemphigoid) OR AB=((Anti-p200 OR "Anti p200" OR "Anti p-200" OR Anti-p-200 OR "Anti p 200"OR "Anti-p 200" ) AND pemphigoid) |
| #3 | TI=(("laminin gamma1" OR "Laminin-gamma1 " OR "laminin gamma-1" OR "laminin-gamma-1" OR "Laminin-gamma 1" OR "Laminin-gamma 1") AND pemphigoid) OR TS=(("laminin gamma1" OR "Laminin-gamma1 " OR "laminin gamma-1" OR "laminin-gamma-1" OR "Laminin-gamma 1" OR "Laminin-gamma 1") AND pemphigoid) OR AB=(("laminin gamma1" OR "Laminin-gamma1 " OR "laminin gamma-1" OR "laminin-gamma-1" OR "Laminin-gamma 1" OR "Laminin-gamma 1") AND pemphigoid) |
| #4 | TI=(("Anti-laminin gamma 1" OR "antilaminin gamma 1" OR "Anti-laminin-gamma 1" OR "Antilaminin-gamma 1" OR "Anti-laminin gamma-1" OR "Antilaminin gamma-1" OR "Anti-laminin-gamma-1" OR "antilaminin-gamma-1" OR "Anti-laminin gamma1" OR "antilaminin gamma1" OR "Anti-laminin-gamma1" OR "Antilaminin-gamma1" ) AND Pemphigoid) OR TS=(("Anti-laminin gamma 1" OR "antilaminin gamma 1" OR "Anti-laminin-gamma 1" OR "Antilaminin-gamma 1" OR "Anti-laminin gamma-1" OR "Antilaminin gamma-1" OR "Anti-laminin-gamma-1" OR "antilaminin-gamma-1" OR "Anti-laminin gamma1" OR "antilaminin gamma1" OR "Anti-laminin-gamma1" OR "Antilaminin-gamma1" ) AND Pemphigoid) OR AB=(("Anti-laminin gamma 1" OR "antilaminin gamma 1" OR "Anti-laminin-gamma 1" OR "Antilaminin-gamma 1" OR "Anti-laminin gamma-1" OR "Antilaminin gamma-1" OR "Anti-laminin-gamma-1" OR "antilaminin-gamma-1" OR "Anti-laminin gamma1" OR "antilaminin gamma1" OR "Anti-laminin-gamma1" OR "Antilaminin-gamma1" ) AND Pemphigoid) |
| #5 | TI=(((200-kd) OR (200 kd) OR (200-kda) OR (200 kda)) AND dermal AND antigen) OR TS=(((200-kd) OR (200 kd) OR (200-kda) OR (200 kda)) AND dermal AND antigen) OR ab=(((200-kd) OR (200 kd) OR (200-kda) OR (200 kda)) AND dermal AND antigen) |
| #6 | #1 OR #2 OR #3 OR #4 OR #5 |
